# A guide for using social media in environmental science and a case study by the Students of SETAC

**DOI:** 10.1186/s12302-015-0062-5

**Published:** 2015-11-23

**Authors:** Sarah R. Bowman, Geert Biermans, Andrea Hicks, Dragan M. Jevtić, Jose Luis Rodriguez-Gil, Erica K. Brockmeier

**Affiliations:** 1 Department of Evolution, Ecology, and Organismal Biology, The Ohio State University, 300 Aronoff Lab, 318 West 12th Avenue, Columbus, OH 43210 USA; 2 Centre for Environmental Research, University of Hasselt, Agoralaan, 3590 Diepenbeek, Belgium; 3 Department of Civil and Environmental Engineering, University of Wisconsin, 2208 Engineering Drive, Madison, WI 53706 USA; 4 Institute of Environmental Sciences, Jagiellonian University, Gronostajowa 7, 30-387 Kraków, Poland; 5 Department of Biological Sciences, University of Calgary, 2500 University Drive NW, Calgary, AB T2N 1N4 Canada; 6 Institute of Integrative Biology, University of Liverpool, Biosciences Building, Crown Street, Liverpool, L69 7ZB UK

**Keywords:** Social media, Science communication, Science outreach, Environmental science, Twitter, Facebook, LinkedIn, Mendeley, ResearchGate

## Abstract

**Background:**

In the past few years, the use of social media has gradually become an important part of our daily lives. While some might see this as a threat to our productivity or as a source of procrastination, social media as a whole have unquestionably changed the way in which information and knowledge disseminate in our society.

**Social media guide:**

This article is meant to serve as a guide for scientists who would like to establish their online presence and includes an outline of the benefits of using social media as well as strategies for establishing and improving your presence in social media. Environmental scientists in particular can benefit enormously from this approach, since this field of science deals with topics that directly impact our daily lives.

**Case study:**

To highlight these approaches for our fellow scientists in the field of environmental science and toxicology and in order to better engage with our own peers, we describe the outreach methods used by the student advisory councils of the Society of Environmental Toxicology and Chemistry (SETAC) and how we have worked towards an improved social media presence. In this article we present our initiatives to increase social media usage and engagement within SETAC. This includes joint social media accounts organized by the SETAC student advisory councils from various SETAC geographical units. We also led a course on social media usage at the SETAC Nashville meeting in 2013 and are currently developing other outreach platforms, including high school student-oriented science education blogs.

**Conclusion:**

The Students of SETAC will continue to increase communication with and among SETAC students on a global level and promote the use of social media to communicate science to a wide variety of audiences.

## Background

As new technologies emerge and the way we receive information changes, so does the way we collect and disseminate scientific knowledge. There has been a significant shift from looking at single articles on library shelves and sharing discoveries only at conferences to now being able to conduct literature reviews on thousands of journals over decades of research using a simple internet search. Data and information sharing among researchers on different continents occurs now in a matter of seconds, and discussions that were previously limited to annual conferences can occur via email on a daily basis. In addition to increased communication, the internet has dramatically increased the visibility of science to the general public [1]. As a result, researchers can receive emails, facebook messages, or tweets from scientists to non-scientists alike with opinions or questions on their results. These communications can include a wide range of curious, fiery, uninformed, mocking, or supportive comments and ideas. These direct discussions between scientists and the general public about the implications of research can, when done in the right way, lead to a better understanding of the message that scientists are trying to convey. This is especially crucial for environmental science due to the global and expansive implications of this work, with its research having impacts on water quality, chemicals in household products, biodiversity, and global climate change. In addition, social media also enables better communication within the scientific community, as a place where Nobel laureates, high school students, and everyone in between can engage in discussions and share ideas on more equal footing than at a conference or in a classroom [2].

Social media are internet-based tools that allow for the free exchange of ideas and content, which transcend the boundaries of geography, analogous to the changes experienced by science as a whole. Social media provide ease of access to people and information, foster transparency of information [3], and have shifted the way in which people gather information [4, 5]. Social networking sites are currently used by 74 % of the adults who have internet access in the United States [6] with the majority of users of a younger age range: 89 % of 18–29 year-olds who use the internet are on social networking sites, followed by 82 % of 30–49 year-olds, 65 % of 50–64 year olds, and 49 % of those 65 and older. No statistically significant variation was seen with respect to gender, educational attainment, or income level. Most social media users can be found on Facebook (71 %), followed by LinkedIn (22 %), Twitter (19 %), and Instagram (17 %). Social media use also varies geographically with the following countries as an example (percentages of adults who use the internet at least occasionally) United States (74 %), Egypt (88 %), Russia (86 %), Turkey (79 %), Brazil (73 %), and China (48 %) [7]. At these usage rates, it is not surprising that social media has changed the way in which people find information.

This number of users and the shift in the way people receive information is easily the most important reason why scientists should pay attention to social media, since people are now using social media services such as Twitter and Facebook in lieu of traditional news outlets as a way to keep up with the large amount of information put forth on an average day [8, 9]. Because of these changes in trends, social media users may be less likely to read a long research manuscript or even a news article and must be drawn quickly towards the essence and importance of the story in order to maintain interest. Because of this, messages need to be clear, precise, and understandable in order to convince the audience to read on and explore the subject. As this is not a skill that is formally taught in the current academic environment, scientists are now learning how to adapt to this new technology with no formal training. Until a significant shift in the way that students and researchers are trained in how to share their information in this forum, scientists are on their own for embracing and effectively using social media.

Scientific researchers have seen the benefits of social media and many are already present on one or more platforms. These include both academic or professional networks such as ResearchGate and LinkedIn as well as more ‘informal’ media such as Facebook and Twitter [10]. While scientists have embraced social media as a professional tool, some are using them only to follow discussions (less than 50 % on Twitter) and even fewer scientists are using these outlets for participating in research discussions or reaching out to new colleagues [10]. In addition, scientists can also use social media as a way to measure the general public’s perception of scientific topics, which can be used to help guide education and outreach programs and to prevent misunderstandings of crucial scientific concepts [11].

Scientists can and are using social media for self-promotion, as a source of information and collaboration, and for outreach [10]. There have been a number of studies in recent years showing positive correlations between article downloads related to social media presence. Some studies suggest that tweeted journal articles can be up to 11 times more likely to be cited as compared to articles without a social media presence [12]. Thanks to social media, scientists have access to a great marketing and promotional tool that can significantly increase the reach of a publication. In addition, recurrent social media presence can help a scientist become recognized as an expert in their field. This obviously can also work the other way: the lack of a virtual presence or the posting of poor content can make even the most senior researcher seem unimportant and can have a lasting negative effect on a scientific career [13].

Social media can also help scientists increase the quality of their interactions with peers. When more scientists use social media to promote their publications, grants, conference presentations, etc., it provides a simple way for others to stay updated and follow-up on research findings that they are interested in. Other researcher-oriented platforms such as ResearchGate and Mendeley can serve as an alternative type of social media that can be used to find other peers working in the same area or with similar research interests. These websites also provide forums for discussion on lab protocols or data analysis methods and allows researchers to ask questions to one another more quickly than by looking for older papers citing complete methodologies.

Scientists who have a direct line of communication from themselves to other scientists to the general public are especially crucial to the environmental sciences. Science outreach is becoming increasingly important for scientists to embrace, as broader impacts and community engagement are important requirements for grants and funding opportunities, so scientists can no longer ignore the need to share their science on a broader level. Many of us work on sensitive, controversial, or impactful issues such as water quality, food security, ecosystem preservation, toxicity of chemicals, etc. Because the general public are increasingly using social media for news on current events and issues [8, 9], it is important for scientists to be clear and present in these venues when they are sharing their work and talking about science in their field. While newspapers and journalists have previously served as a link between environmental researchers and the public, this method can lead to misinterpretations, omissions, and sensationalism depending on the way the message is portrayed. Social media therefore provides scientists an opportunity to share their research without a ‘middle man’ while still providing the public a chance to engage in discussion about it.

When social media are used as a dual-purpose communication outlet, as outreach and information sharing for the public, and as a way to share papers and findings with colleagues, social media can serve as a source of inspiration and as a point of contact for researchers. The more passive nature of information-gathering on social media facilitates finding new research and colleagues that would have been less accessible in the past. In this discussion paper, we provide a guide for environmental scientists for establishing and improving social media presence. We then present the initiatives of the Students of SETAC as a case study to highlight the steps laid out by the guide.

## A guide for establishing and improving your social media presence

Since social media is a communication tool and not a communication goal, there are many ways of using social media depending on what you want out of it. Scientific social media usage without a clear vision of what the message is and how it should be presented is like holding a megaphone and shouting whatever crosses your mind. Because social media give a direct contact to the public, researchers using this tool should be fully aware of the potential impact of messages posted using these forums. Below are our recommended six steps for establishing and improving your social media presence as an environmental scientist:

1. *Define your goals*. What message do you want to deliver? Who will be your audience? Do you want to talk to the public about how important your work is or focus on keeping your peers informed about your research? Do you want to be a general source of information about your particular field or to provide insights to the general public about broader scientific issues? Answers to these questions are certainly not binding and you can evolve your message through time, but at the beginning of your time as a social media user, it is important to set specific goals and to work towards them.

2. *Be present, observe, and learn*. The best way to learn how a particular social network works is to make use of it in your personal life. A large number of young scientists are already present on one or more social media outlet, especially Facebook or Twitter. For scientists not yet present on any social media or on a certain network, the important thing is to make a profile. This does not have to be your final ‘professional’ profile but should allow you to ‘feel’ what the specifics of the medium are in order to learn how it functions. This also allows you to see firsthand its pitfalls and drawbacks.

For example, Facebook is a very efficient network for sharing content and for building thematic pages for a wide audience, but if certain comments are left unaddressed, negative or uninformed discussions can take a sharp turn for the worst. As another example, Twitter uses short messages and has a very efficient sharing system (re-tweets) but requires frequent postings to compete with the volume of other posts in order to be at the top of a newsfeed. Twitter is also great for conferences and networking at meetings, as it enables you to participate in conversations and learn about trending topics at large annual meetings where you may not be able to attend every session. However, Twitter posts are by default public, so caution must be exerted in what you say using this platform [14]. Other networks such as ResearchGate or Mendeley are focused on finding peers and research articles but do not necessarily enable a wider debate among a general audience.

The key with this step is to experience the different types of social media networks, see their attributes from a professional angle, and determine which fit your goals best. Think about how you could combine several to get the audience you want. It is also a good idea to observe how other popular and well-followed scientists deliver their message to their colleagues and the public and what styles they use to communicate using various types of media.

3. *Think big but start small.* Once you have identified your message, your goals, and your target network(s), you can start to build your social media presence. You can either divert your personal or previously established profile towards a more scientific angle or start from scratch to build a new or parallel professional account. This choice should be based on whether you want to mix your professional and personal message and style. The important thing to consider here is to be consistent in the type of content and timing of posts that you share on your timeline or page. A typical approach would be a researcher who has a scientific blog and expands the reach of his posts by linking them to Facebook, Twitter, ResearchGate or LinkedIn. Each platform allows for different types of sharing and content engagement. There is a learning curve to this, and some strategies may not pay off, but the benefit of social media is that you can always adapt and modify your strategy at any time with little effort.

4. *Find and engage with your audience.* This step is platform-specific, as each type of social media will differ in how you find new followers and engage with your current followers. For example, Twitter has the ‘lists’ feature, which allows users to curate lists of users that tweet on a specific subject or hashtags. Lists allow a user to find tweets and (other users to interact with) on a certain topic. ResearchGate and Facebook also have tags and categories that can help users find peers or interesting pages. Keep in mind that a good audience is built slowly and through interaction, so the best approach is not to overreach beyond your target audience or resort to overly aggressive advertising. At the end of the day, a valuable and well-thought message will attract a valuable audience on its own, requiring only a little patience accompanied by quality and well-timed content.

5. *Enhancing your connections.* When your online presence has been established and you have begun to add content to your page, you should continue to work on improving your online presence. This includes maintaining your rate and quality of posts to make sure that your followers do not lose interest in your page. For social media pages that allow for engagement with your followers (especially Facebook comments), be sure to respond to the questions and comments of your followers and to engage with the people that are interested in your content (including those with negative comments). To some extent, your page’s popularity will increase organically (i.e. people sharing the page with interested colleagues) but some level of advertising is also required. If you intend to share work with new colleagues or collaborators, conferences and workshops are a great place to highlight your social media pages. For the general public, joining in on larger conversations on popular topics in the news is a good start towards generating future interest in your specific line of work.

6. *Refine your message* and *explore new platforms.* As you continue to use social media, be sure to work to keep your message fresh and in line with current trends. Use feedback from your followers such as comments, re-tweets, and post-sharing to figure out which types of posts are most popular and if there is a certain type of content that seems to be the most engaging. Pictures instead of large blocks of text are generally more popular and lead to more engagement from your followers, while a short overview of a blog post followed by a link can lead to more clicks than a link with no description. With platforms such as Twitter, hashtags can allow you to enter into wider conversations, join in on trends, and to find ways to share your message with a larger audience. In addition, be on the look-out for emerging trends and new forms of social media and be open to trying something new to share your message. Setting up a Vine or Instagram account for your lab may not make sense at first thought, but being creative in finding other visual ways to share your science with videos or photos could make all the difference in expanding your online presence.

## Students of SETAC social media case study

‘Students of SETAC’ is the term adopted to represent the SETAC North America student advisory council (NASAC), the SETAC Europe student advisory council (SAC Europe), and other SETAC regional and geographical units’ student representatives as a collective unit. Under this mantra we set up mutually-operated forums for reaching out to other environmental chemistry and toxicology students using Twitter, Facebook, and LinkedIn. In addition to our specific goals indicated below, we believe that social media can benefit SETAC and its students by enabling connections not previously available. Using social media platforms, we can better connect SETAC students to jobs and fellowship opportunities, highlight the high-quality science done by our student members, and provide a forum for students to interact during the entire year, not just at an annual meeting.

**Fig.1:**
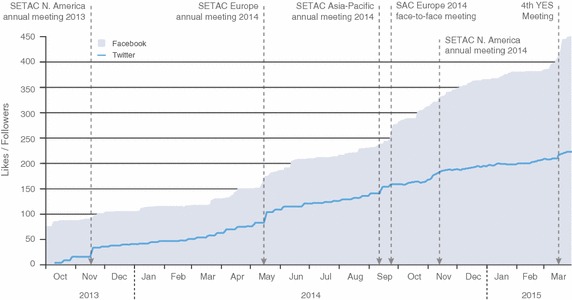
Number of likes to the Students of SETAC Facebook Page (*shaded area*) and followers of the @StudentsofSETAC Twitter account (*solid line*). *Vertical dashed arrows* mark significant events through the year during which the number of likes/followers peaked

**Fig.2:**
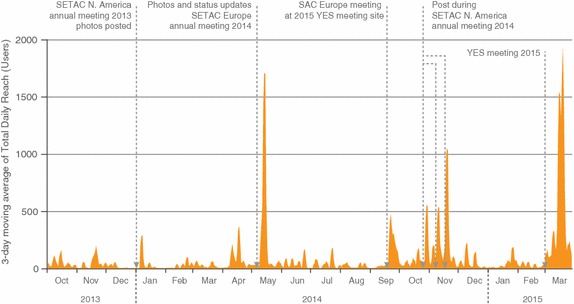
Three-day moving average of the total daily reach (number of unique users who have seen any content associated with the Page) for the Students of SETAC Facebook Page from the initiation of the page (September 2013) through March 2015. *Arrows* mark events and activities through the year with peaks in post reach

**Fig.3:**
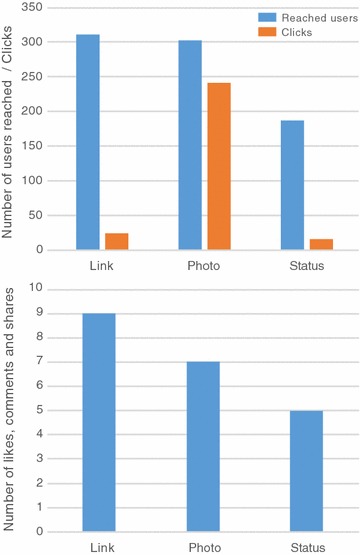
Average reach (number of unique users who have seen any content associated with the page) and clicks by users in posted links, pictures or status updates (*top*), and average number of likes, comments and shares (*bottom*) of different post types of the Students of SETAC Facebook Page

1. *Define your goals.* The primary goals for our Students of SETAC social media efforts are to increase our online presence as student members of SETAC, update students on issues, jobs, and opportunities relevant for environmental toxicology and chemistry, and create a forum for increased discussion and engagement by our student members. The primary audience of our present social media platforms includes students and recent graduates in the fields of environmental toxicology and chemistry. With this goal and audience in mind, we launched our Students of SETAC Facebook Page [15] in September 2013. As we slowly built our Facebook presence, our followers (measured as the number of page ‘likes’) grew steadily and the page now has over 500 likes (Fig. 1).

2. *Be present, observe, and learn.* Since creating the Facebook Page, we have been monitoring the total daily reach (number of unique users who have seen any content associated with the page).Total daily reach is one way to measure user engagement on Facebook Pages. Figure 2 demonstrates our post reach since the inception of our Facebook Page. Overall, we recorded the greatest post reach when content from face-to-face events was posted such as after annual conferences (Fig. 2). In general, our greatest interaction comes from posts involving photos and links (Fig. 3). We have more page views when links and photos are posted (Fig. 3) and continued usage of the page (measured by the number of clicks) with photo posts. While not as prominent as photos and links, status updates, which can include announcements on job postings, SETAC-related opportunities, events, professional training courses, meetings, and upcoming annual meeting student events, are also resulting in strong page engagement (Fig. 3). By monitoring our engagement with users, we are able to recognize the most preferred types of posts (photos and links) so that we can use those in the future to continue reaching our target audience.

With over 200 followers (around 12 % of our potential audience), our Twitter account (@Students of SETAC) follows a similar trend, with large peaks of the number of followers during or immediately following the meetings of our different geographic areas (Fig. 1). International coverage of our Twitter followers appears to expand slower than it does for Facebook. About 52 % of our followers are located in North America (34 % USA, 18 % Canada), 20 % in Europe and 5 % in Australia (an additional 20 % is covered by other countries or did not disclose their location). Due to this geographic distribution, English is the majority language (88 % of followers). Several recent communications, however, have now been sent in other languages, including Spanish and French.

3. *Think big but start small.* Our current number of Facebook followers represents approximately 25 % of our potential audience based on global SETAC student membership. With the primary SETAC Facebook Page covering 41 % of total membership since their page launch in early 2012, we consider our growth in just a year and a half to be at a pace that can easily reach and even surpass that of SETAC’s page in the coming year. We made sure to develop our presence on Twitter and Facebook and to ensure the regular posting of content for our followers before expanding into other social media outlets, including LinkedIn, and are now using the momentum gained from our social media accounts as we look to developing a Students of SETAC blog.

In addition to increasing the types of social media platforms we use, we have worked to develop our extensive geographical reach which includes students from all across the world. We achieve this by having geographical representation of the Students of SETAC posting team, which includes student members from Europe, North America, Australia, and South America. Currently, the most engaged geographical group is Serbia, which was the location of the 2015 YES meeting, and the most engaged age group is 25–34 years old. According to the National science foundation survey of earned doctorates, the average age at doctoral completion for the life sciences field was 31.8 in 2013 with an average completion time (from the start of graduate school) of 6.9 years [16]; these data clearly show that we are reaching our target audience of (graduate) students and recent graduates.

4. *Find and engage with your audience.* On our Facebook Page, our social media account managers interact with our followers by sharing relevant posts to our page as well as replying to comments and messages on a regular basis. Twitter has provided us with some of the best opportunities to interact with our audience, as the use of hashtags (#) during SETAC meetings allows for the “crowdsourcing” of the contents we provide by allowing our followers to post from their own accounts while maintaining a common thread that allows everyone else to be involved in the discussions. #SETAC Basel was used during the SETAC Europe meeting in Basel in May 2014 at a SETAC event. This was followed by a very successful online presence at the #SETAC Vancouver meeting in November 2014, with the Students of SETAC account having several featured tweets during the meeting. The hashtag, #SETAC YES, was successfully used leading up to and during the 2015 YES meeting, and we will continue to use this hashtag for future SETAC YES conferences.

We are also using social media to engage in larger conversations with SETAC students at conferences. At the SETAC Vancouver meeting we began asking students the question “What does SETAC mean to you?,” which was followed up with a workshop at the SETAC YES meeting and as a tweet-up at the SETAC Barcelona meeting. A Q&A session was held via Twitter at the SETAC Barcelona meeting about the benefits of becoming involved in the Society. The tweet-up included SETAC members that were present in Barcelona, but also involved others who could not make it to the conference. We will continue to initiate and engage in larger conversations with SETAC students at the upcoming SETAC Salt Lake City annual meeting in November 2015.

5. *Enhancing your connections.* As the Students of SETAC, we have successfully built and now currently maintain Facebook and Twitter platforms to communicate with each other. Even with changes in SETAC regional member representation, our team keeps up with regular posts and we continue to advertise our page at annual and regional meetings to new SETAC students. We will soon host the first North American SETAC YES meeting in February 2016, which will be an opportunity to expand on the content associated with the #SETACYES hashtag and to continue conversations about the benefits of being involved in the Society.

The Students of SETAC are also at the forefront of enhancing the online presence of the Society as a whole by working with the SETAC office. At the SETAC Vancouver meeting, SETAC and NASAC members facilitated a Reddit Ask Me Anything while recruiting scientists at the meeting to answer questions fielded by this online community. We will continue to explore this forum at future SETAC meetings as a way to better link our conferences to public outreach activities.

6. *Refine your message and explore new platforms.* As we continue to evaluate what types of content our followers like to see on our pages, we are also looking ahead to future goals for our group. Our next social media initiative will focus on communicating with new audiences, particularly younger students. Our first initiative is outreach to students using a Students of SETAC blog. Our audience will be high school and undergraduate students interested in environmental science. The goal of the blog is to communicate what it is like to be a graduate student/scientist with the hopes that the blog will get younger students interested in environmental science. This blog will include posts by Students of SETAC about their research and day-to-day activities in the lab. The blog will include dialogue boxes where high school and undergraduate students can ask the researcher questions to create a back-and-forth dialogue about the topics. The blog will also incorporate engaging videos such as lab tours and field work demonstrations. While the blog is still currently under development (December 2015 target release), we have worked on our ideas with high school teachers in order to understand the most classroom-friendly designs and types of content.

Other Students of SETAC social media-related initiatives have also included a social media professional training course at the 2013 SETAC North America annual meeting in Nashville, Tennessee. This course was proposed as an idea and supported by the SETAC North America Board of Directors and was taught by members of NASAC and SAC Europe. The structure of the course followed the main points of our guide for using social media. We discussed specific platforms including Facebook, ResearchGate, LinkedIn, Twitter, and Mendeley. We will now focus future efforts on formatting the professional training course as a SETAC webinar, online course, or noon-time seminar. As SETAC continues to integrate social media into annual meeting activities, members will likely continue to recognize the importance of using social media for science communication among themselves and with the public.

## Conclusions

In an increasingly global world which is overflowing with information, different social media platforms have the strong potential to promote scientific results and achievements, connect with peers, and interact with the general public. Environmental scientists in particular should use this opportunity due to the impact of this type of research which connects society to so many important parts of our world, from the air we breathe to the foods and products we bring into our homes. The initiatives by the Students of SETAC provide an example of how a strong and active social media presence with clearly defined outreach goals can be used to disseminate information and reach a target audience. We propose that this guide for using social media in the environmental sciences can be adopted and used by different research groups, laboratories, and other types of science-related societies and communities.

## Abbreviations

NASAC: SETAC North America Student Advisory Council
SAC Europe: SETAC Europe Student Advisory Council
SETAC: Society of Environmental Toxicology and Chemistry
YES: Young Environmental Scientists

## References

[1] Ynalvez MA, Duque RB, Shrum W, Dutton WH, Jeffreys PW (2010). Shaping research in developing areas. World wide research: reshaping the sciences and humanities.

[2] Claussen JE, Cooney PB, Defilippi JM, Fox GM, Glaser SM, Hawkes E, Hutt C, Jones MH, Kemp IM, Lerner A, Midway SR, Nesbit S, Osborne-Gowey J, Roberts R, Steward C (2013). Science communication in a digital age: social media and the American fisheries society. Fisheries.

[3] Afshar V (2013) Five ways social media has forever changed the way we work. Huffington post. http://www.huffingtonpost.com/vala-afshar/social-media_b_2944407.html. Accessed 24 May 2013

[4] Fraidaki K, Pramatari K, Doukidis G (2014) Living in the era of social media: how the different types of social media may affect information acquisition process. In: Meiselwitz G (ed) Social computing and social media. Lecture notes in computer science.vol 8531. Springer, Berlin, pp 178–185

[5] Stephens M, Yoo J, Mourao RR, Vu HT, Baresch B, Johnson TJ (2014). How app are people to use smartphones, search engines, and social media for news?: examining information acquisition tools and their influence on political knowledge and voting. J Inform Tech Polit.

[6] Pew research center (2014) Social networking fact sheet. http://www.pewinternet.org/fact-sheets/social-networking-fact-sheet. Accessed 19 Sep 2014

[7] Rainie L, Jacob P (2014) Emerging nations catching up to U.S. on technology adoption, especially mobile and social media use. Pew Research Center. http://www.pewresearch.org/fact-tank/2014/02/13/emerging-nations-catching-up-to-u-s-on-technology-adoption-especially-mobile-and-social-media-use/. Accessed 13 Feb 2014

[8] Hermida A, Fletcher F, Korell D, Logan D (2012). Share, like, recommend: decoding the social media news consumer. J Stud.

[9] Kwak H, Lee C, Park H, Moon S (2010) What is Twitter, a social network or a news media? Proceedings of the 19th international conference on world wide web, pp 591–600

[10] Van Noorden R (2014). Online collaboration: scientists and the social network. Nature.

[11] Roberge J-M (2014). Using data from online social networks in conservation science: which species engage people the most in Twitter?. Biodivers Conserv.

[12] Eysenbach G (2011). Can tweets predict citations? Metrics of social impact based on Twitter and correlation with traditional metrics of scientific impact. J Med Internet Res.

[13] Bik HM, Goldstein MC (2013). An introduction to social media for scientists. PLoS Biol.

[14] Tachibana C (2014). A scientist’s guide to social media. Science.

[15] Students of SETAC Facebook Page. http://www.facebook.com/studentsofSETAC

[16] National Science Foundation: Doctorate recipients from U.S. Universities (2013) National Center for Science and Engineering Statistics and Directorate for Social, Behavioral Economic Sciences. NSF 15–304. http://www.nsf.gov/statistics/sed/2013/digest/index.cfm. Accessed 5 Aug 2015

